# Supplementary figures and images for: Bacterial and archaeal spatial distribution and its environmental drivers in an extremely haloalkaline soil at the landscape scale
Source: PeerJ. 2019 Jun 18;7:e6127. doi: 10.7717/peerj.6127 (PMC6587938; doi:10.7717/peerj.6127)

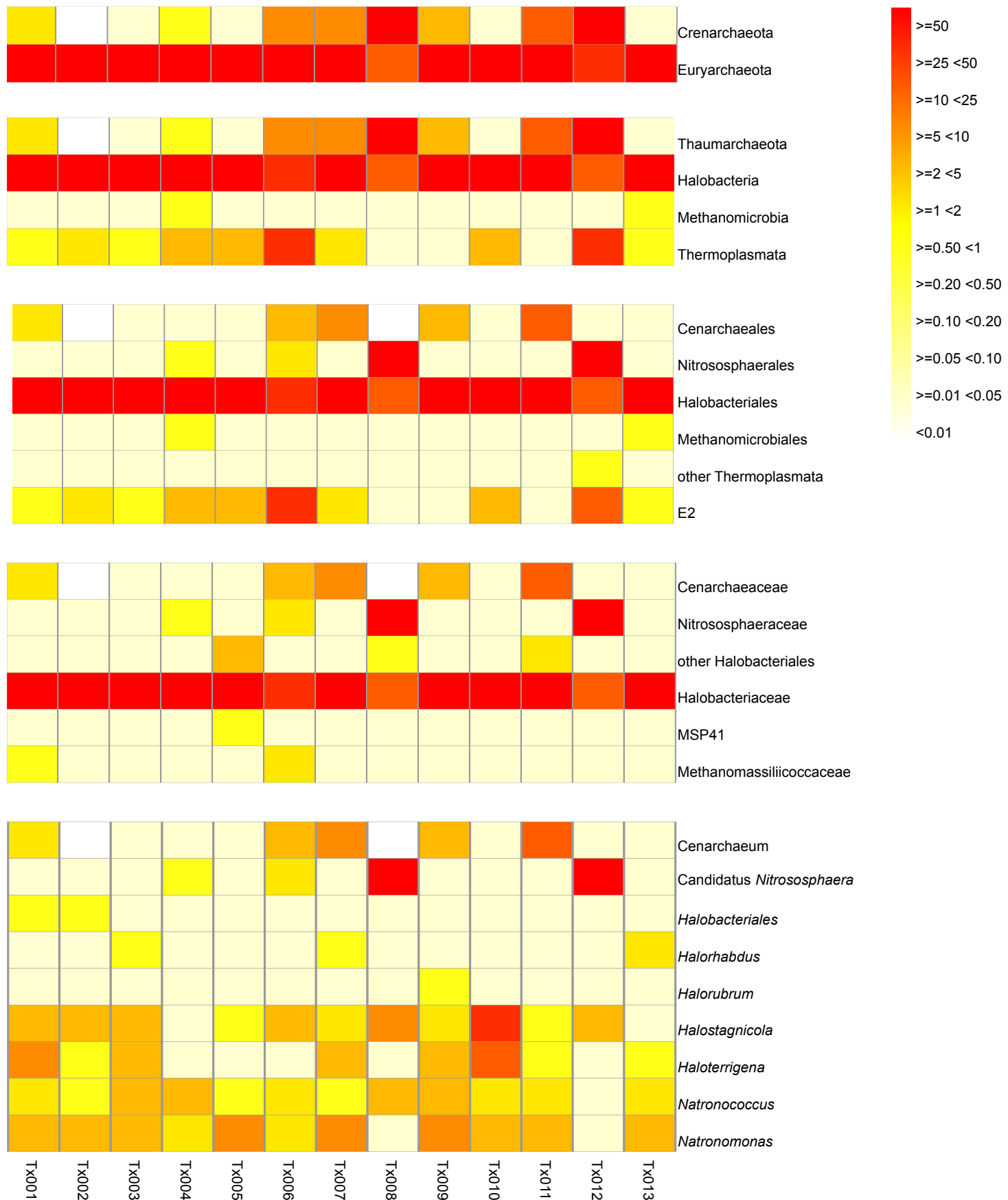

Supplement: Supplemental Information 1 — Heatmap with the relative abundance (%) of Archaea at different taxonomic levels along a southeast sampling transect (Tx001-Tx013) in soil of the former lake Texcoco. Each sampling site is referred with the key Tx followed by consecutive number indicating progression in spatial separation. [file peerj-07-6127-s001.pdf]

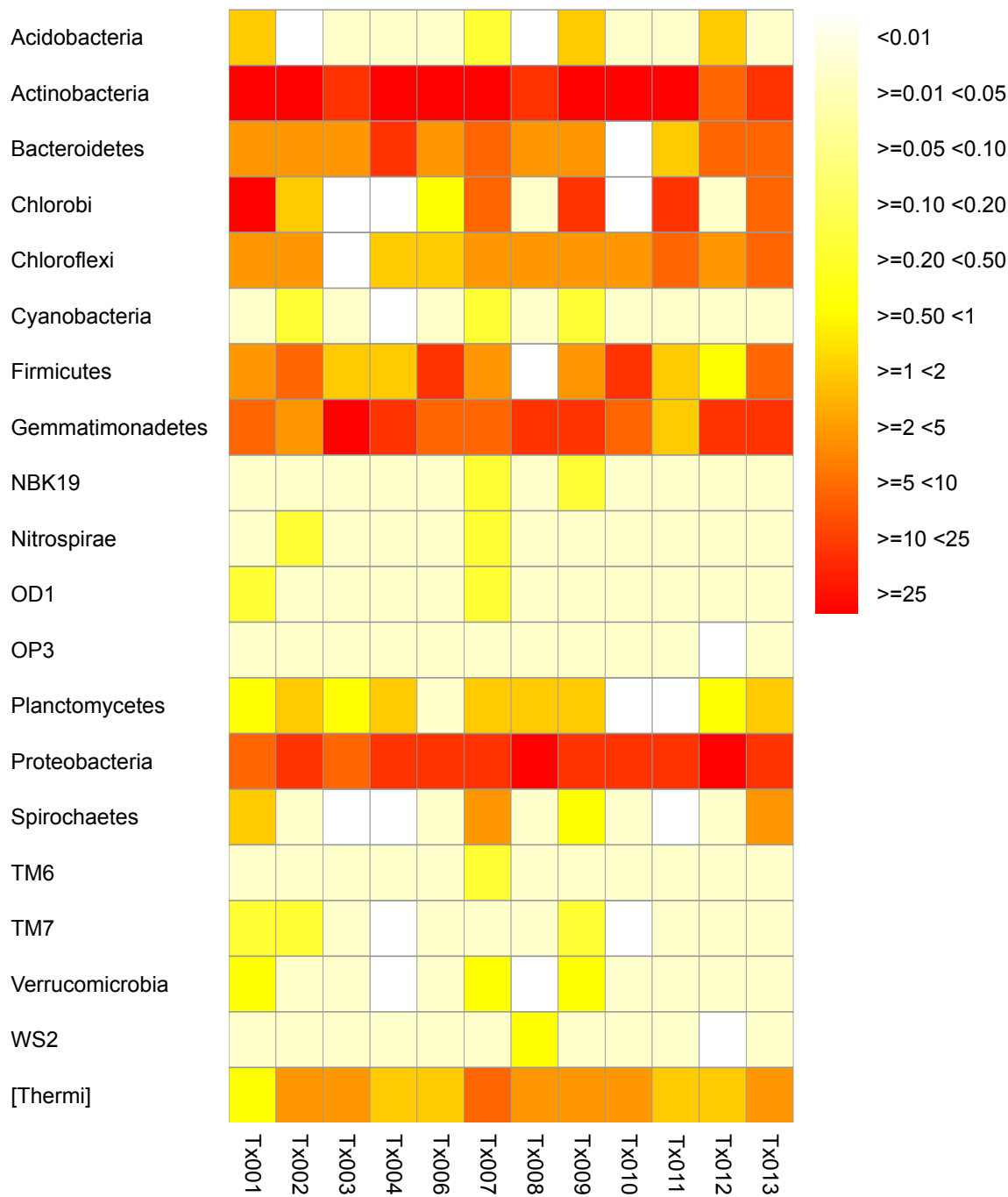

Supplement: Supplemental Information 2 — Heatmap with the relative abundance (%) of bacterial phyla along a southeast sampling transect (Tx001-Tx013) in soil of the former lake Texcoco. Each sampling site is referred with the key Tx followed by consecutive number indicating progression in spatial separation. [file peerj-07-6127-s002.pdf]

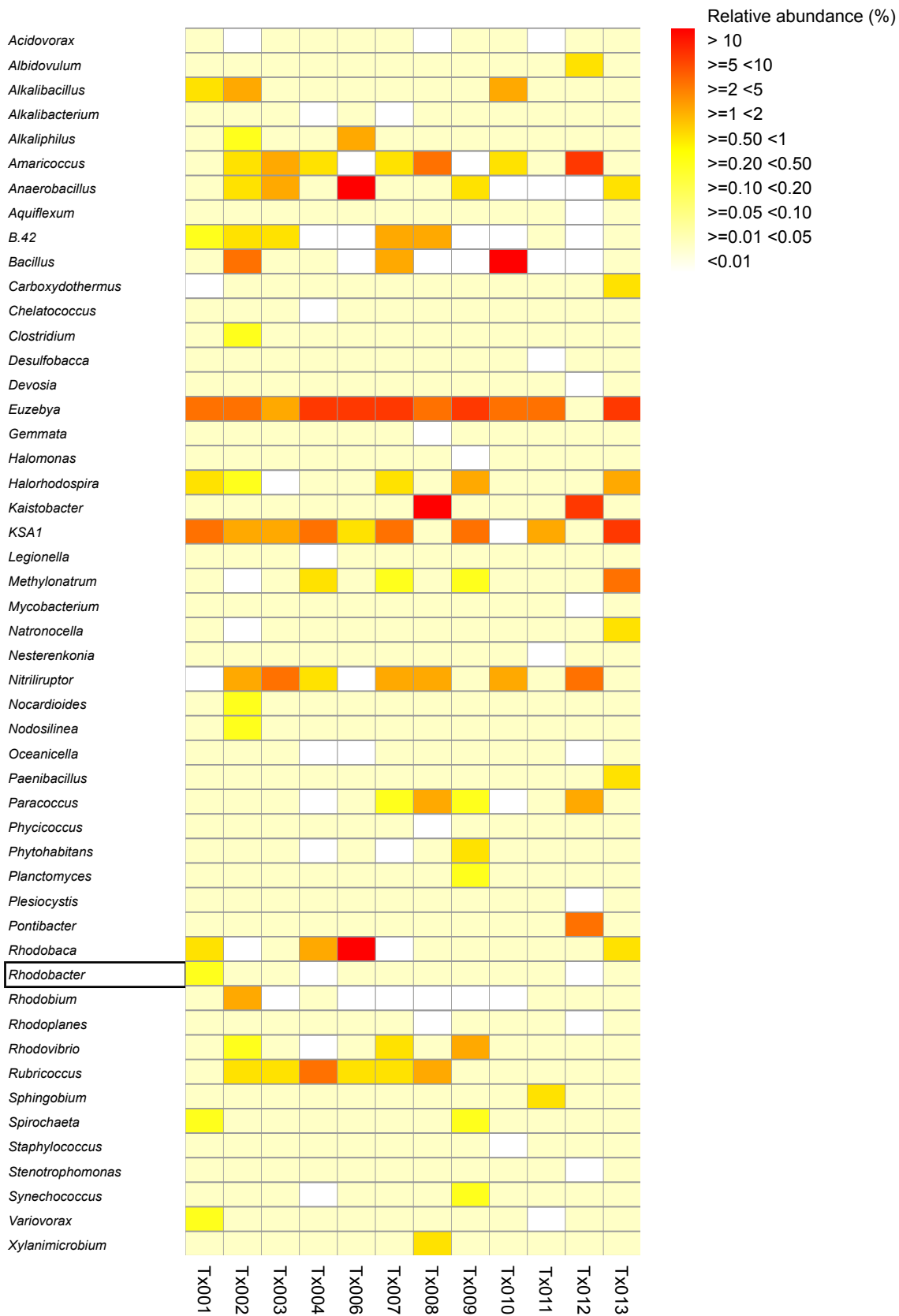

Supplement: Supplemental Information 3 — Heatmap with the relative abundance (%) of bacterial genera along a southeast sampling transect (Tx001-Tx013) in soil of the former lake Texcoco. Each sampling site is referred with the key Tx followed by consecutive number indicating progression in spatial separation. [file peerj-07-6127-s003.pdf]

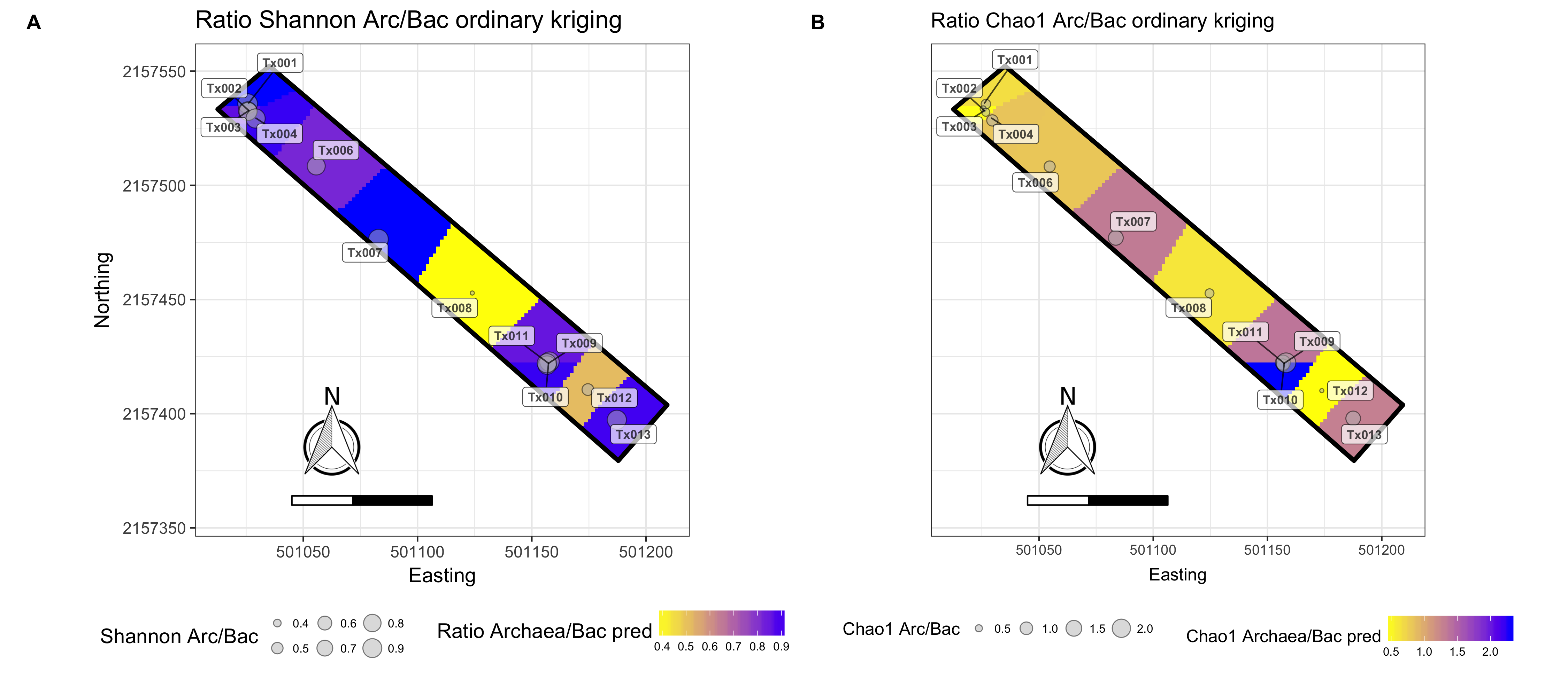

Supplement: Supplemental Information 4 — (A) Shannon diversity ratio Archaea/Bacteria and (B) Chao1 species richness ratio Archaea/Bacteria. Pred: predicted values of diversity indexes by ordinary kriging on a 3000-pixel grid (2.38 m2 each); Obs: observed values of diversity indexes at transect sampling sites (Tx001-Tx013). The coordinates are in UTM (14N). [file peerj-07-6127-s004.png]
