# Supplementary material for: Bacterial and archaeal spatial distribution and its environmental drivers in an extremely haloalkaline soil at the landscape scale
Source: PeerJ. 2019 Jun 18;7:e6127. doi: 10.7717/peerj.6127 (PMC6587938; doi:10.7717/peerj.6127)
Supplement: Supplemental Information 5 — Soil description for each sampling site set at irregular distances along a southeast-running transect at the Texcoco former lake bed area and descriptive statistics analysis for each measured parameter on the whole. Each sampling site is referred with the key Tx followed by consecutive number indicating progression in spatial separation. The sampling was done on August 22, 2014. [file peerj-07-6127-s005.docx]

| Site | Localization | pH | WC^a^  (%) | EC^b^  (dS m^-1^) | Inorganic C | Organic C^d^ | | Sand  (g kg^-1^) | Slit  (g kg^-1^) | Clay  (g kg^-1^) | Textural classification |
| --- | --- | --- | --- | --- | --- | --- | --- | --- | --- | --- | --- |
|  |  |  |  |  | (g kg^-1^ ds^c^) | | |  |  |  |  |
| Tx-001 | 19d30'45 N  98d29'24.8W | 10.4 | 55.52 | 66.5 | 6.8 | | 17.4 | 616 | 127 | 256 | Sandy clay loam |
| Tx-002 | 19d30'44.9 N  98d29'24.8W | 10.5 | 42.24 | 84.1 | 8.2 | | 15.0 | 510 | 168 | 322 | Sandy clay loam |
| Tx-003 | 19d30'44.9 N  98d29'24.8W | 10.6 | 43.56 | 45.1 | 5.3 | | 10.0 | 471 | 240 | 290 | Sandy clay loam |
| Tx-004 | 19d30'44.8 N  98d29'24.7W | 10.5 | 47.33 | 81.2 | 7.4 | | 17.0 | 511 | 157 | 333 | Sandy clay loam |
| Tx-005 | 19d30'44.5 N  98d29'24.3W | 10.5 | 30.17 | 142 | 6.0 | | 9.2 | 338 | 315 | 346 | Loamy clay |
| Tx-006 | 19d30'44.1 N  98d29'23.8W | 10.5 | 33.22 | 46.7 | 3.7 | | 5.7 | 611 | 175 | 214 | Sandy clay loam |
| Tx-007 | 19d30'43.1 N  98d29'22.8W | 10.5 | 49.50 | 69.3 | 7.6 | | 11.3 | 562 | 159 | 280 | Sandy clay loam |
| Tx-008 | 19d30'42.3 N  98d29'21.4W | 10.6 | 19.47 | 18 | 5.8 | | 4.5 | 549 | 259 | 192 | Sandy Loam |
| Tx-009 | 19d30'41.3 N  98d29'20.3W | 10.5 | 43.76 | 129.8 | 11.4 | | 12.5 | 546 | 162 | 292 | Sandy clay loam |
| Tx-010 | 19d30'41.3 N  98d29'20.3W | 10.6 | 27.54 | 179.8 | 6.3 | | 12.6 | 390 | 304 | 307 | Loamy clay |
| Tx-011 | 19d30'41.3 N  98d29'20.2W | 10.4 | 47.18 | 113.3 | 8.1 | | 15.4 | 597 | 109 | 294 | Sandy clay loam |
| Tx-012 | 19d30'40.9 N  98d29'19.7W | 10.3 | 13.33 | 7.7 | 5.1 | | 12.5 | 331 | 350 | 319 | Loamy clay |
| Tx-013 | 19d30'40.5 N  98d29'19.3W | 10.4 | 50.67 | 145.9 | 10.0 | | 26.3 | 526 | 199 | 275 | Sandy clay loam |
| Min |  | 10.31 | 13.33 | 7.7 | 3.7 | | 4.5 | 331 | 109 | 192 |  |
| Max |  | 10.62 | 55.52 | 179.8 | 11.4 | | 26.3 | 616 | 350 | 346 |  |
| Var^e^ |  | 0.009 | 165.97 | 2736.5 | 31.1 | | 43.56 | 932.6 | 589.0 | 198.6 |  |
| SD^f^ |  | 0.095 | 12.88 | 52.3 | 2.1 | | 5.6 | 96.6 | 76.7 | 44.6 |  |
|  | | | | | | | | | | | |
