# Supplementary material for: Bacterial and archaeal spatial distribution and its environmental drivers in an extremely haloalkaline soil at the landscape scale
Source: PeerJ. 2019 Jun 18;7:e6127. doi: 10.7717/peerj.6127 (PMC6587938; doi:10.7717/peerj.6127)
Supplement: Supplemental Information 6 — Selected fitting model, Sill, range nugget effect and nugget/sill relation of semivariograms, were estimated using Gstat package in R environment. [file peerj-07-6127-s006.docx]

| Parameter | Model | Sill | Range (m) | Nugget | Nugget/Sill ratio |
| --- | --- | --- | --- | --- | --- |
| pH | Periodic | 0.008 | 40 | 0.003 | 0.375 |
| EC^a^ | Periodic | 2500 | 40 | 500 | 0.200 |
| WC^b^ | Periodic | 250 | 73 | 10 | 0.04 |
| Ci^c^ | Periodic | 2.9 | 50 | 0.5 | 0.172 |
| Co^d^ | Periodic | 28.6 | 55 | 0.8 | 0.028 |
| Clay | Periodic | 36 | 42.5 | 3 | 0.083 |
| Sand | Periodic | 75 | 42.5 | 20 | 0.267 |
| Silt | Periodic | 70 | 43 | 5 | 0.071 |
