# Supplementary material for: Bacterial and archaeal spatial distribution and its environmental drivers in an extremely haloalkaline soil at the landscape scale
Source: PeerJ. 2019 Jun 18;7:e6127. doi: 10.7717/peerj.6127 (PMC6587938; doi:10.7717/peerj.6127)
Supplement: Supplemental Information 7 — A cross validation was done on each of bacterial or archaeal abundances and soil properties using the gstat package in the R environment. The most representative phyla and soil characteristics are presented (nFold = 13). [file peerj-07-6127-s007.docx]

| **Factor** | **ASE^a^** | **RMSE^b^** | **RMSEr^c^** |
| --- | --- | --- | --- |
| pH | 0.072 | 0.085 | 0.889 |
| EC^d^ | 42.048 | 32.653 | 0.624 |
| WC^e^ | 15.669 | 14.269 | 1.108 |
| Ci^f^ | 2.301 | 1.202 | 0.576 |
| Silt | 6.892 | 4.058 | 0.529 |
| Clay | 3.196 | 6.143 | 1.378 |
| Archaea Shannon | 0.708 | 0.599 | 0.534 |
| Archaea Chao1 | 39.059 | 46.430 | 0.626 |
| Bacteria Shannon | 0.374 | 0.201 | 0.438 |
| Bacteria Chao1 | 129.360 | 34.564 | 0.280 |
| Arch/Bac^g^ Shannon | 0.108 | 0.098 | 0.530 |
| Arch/Bac Chao1 | 0.348 | 0.479 | 0.892 |
| Euryarchaeota | 0.163 | 0.301 | 1.177 |
| Thaumarchaeota | 0.167 | 0.289 | 1.107 |
| Proteobacteria | 0.097 | 0.035 | 0.306 |
| Actinobacteria | 0.131 | 0.091 | 0.771 |
| Bacteroidetes | 0.029 | 0.013 | 0.448 |
| Chlorobi | 0.170 | 0.027 | 0.349 |
| [Thermi] | 0.012 | 0.013 | 0.922 |
| Firmicutes | 0.061 | 0.099 | 1.615 |
|  | | | |
