# Supplementary material for: Bacterial and archaeal spatial distribution and its environmental drivers in an extremely haloalkaline soil at the landscape scale
Source: PeerJ. 2019 Jun 18;7:e6127. doi: 10.7717/peerj.6127 (PMC6587938; doi:10.7717/peerj.6127)
Supplement: Supplemental Information 8 — Euclidean distances of scaled environmental variables that have the maximum (rank) correlation with community dissimilarities (BIOENV function from vegan package in R). BIO-ENV Spearman rank coefficients (r) for correlation between soil bacterial and archaeal communities, and physicochemical soil properties. [file peerj-07-6127-s008.docx]

|  | **Parameter subset** | **r Coefficient** |
| --- | --- | --- |
| Archaeal Communities Phylum | pH WC^a^ EC^b^ Clay | 0.6386146 |
| Archaeal Communities Genera | pH WC Clay | 0.6133534 |
| Bacterial Communities Phylum | WC EC Silt | 0.5359622 |
| Bacterial Communities Genera | pH WC Sand Clay | 0.6764083 |
|  | | |
|  | | |
